# A Case of Necrosis of the Superior Maxillary Bone

**Published:** 1891-01

**Authors:** B. D. Friedenwald


### A CASE OF NECROSIS OF THE SUPERIOR MAXILLARY BONE.

BY B. D. FRIEDENWALD, D.D.S.

Necrosis of the superior maxilla is rare. As I have had such a case under my treatment, I think it may be of sufficient interest to warrant its publication.

About three months ago a young woman applied to me for treatment, and I found her suffering from alveolar abscess. After the usual symptoms being manifested, fluctuation was detected. A free incision was made with a scalpel and quite a quantity of pus flowed through the opening, much to the patient's relief.

The superior left central incisor, which was the offending cause, was then extracted, and the patient, who was in an extremely anæmic condition, was put on tonic treatment.

The opening made by the scalpel quickly healed, and the patient seemed entirely well. About four weeks later she again applied to me for treatment, complaining of severe pain about the seat of the former abscess.

A horribly offensive odor was issuing from the mouth, and on making an examination a sinus was discovered. On probing, necrosed bone was found. Fearing that, unless treatment was decided upon, the patient would again suffer with alveolar abscess, I decided to operate.

All the instruments to be used were boiled and placed in a solution of carbolic acid. The patient's face and mouth were washed with a solution of bichloride of mercury. My own hands were disinfected with a similar solution.

The patient was then anæsthetized with chloroform. By means of a circular engine-saw and a surgical engine a free opening was made on the outer side of the right central and the left lateral incisor teeth almost an inch deep. The bone and tissue were then cut away by means of bone-forceps.

The wound was then washed out and disinfected with a solution of bichloride of mercury. A dressing of carbolized lint was then packed into the opening and secured by ligatures to the adjacent teeth. This dressing was changed twice daily for one week, when it was removed entirely.

A mouth-wash, consisting of boracic acid, ten grains to the ounce of water, was given, to be used every hour. In two weeks after the operation the wound had healed, and there was little or no soreness.

An impression of the mouth was then taken and a plate made, with a left superior central incisor attached to it, together with a piece of rubber running up into the space left by the removal of the necrosed bone.

So far the patient has felt no uneasiness, and I have every right to believe that the operation has been a success.

